# Clinical parameters of hypervirulent *Klebsiella pneumoniae* disease and ivermectin treatment in New Zealand sea lion (*Phocarctos hookeri*) pups

**DOI:** 10.1371/journal.pone.0264582

**Published:** 2022-03-03

**Authors:** Sarah A. Michael, David T. S. Hayman, Rachael Gray, Wendi D. Roe

**Affiliations:** 1 Sydney School of Veterinary Science, The University of Sydney, Camperdown NSW, Australia; 2 School of Veterinary Science, Massey University, Palmerston North, New Zealand; 3 Molecular Epidemiology and Public Health Laboratory, Hopkirk Research Institute, Massey University, Palmerston North, New Zealand; Animal Health Centre, CANADA

## Abstract

Hypervirulent *Klebsiella pneumoniae* infection causes significant mortality of endangered New Zealand sea lion pups at Enderby Island, Auckland Islands. Gross necropsy and histopathology findings are well reported, but little is known about the clinical course of disease in affected pups. To determine factors feasible as clinical screening tools for hypervirulent *K*. *pneumoniae* in live pups, 150 pups over two field seasons (2016–18) were recruited shortly after birth for a prospective cohort study. A randomised controlled clinical treatment trial with the anthelmintic ivermectin was conducted concurrently and risk factor data and biological samples were collected approximately fortnightly. Treatment with ivermectin has been demonstrated to reduce the risk of hypervirulent *K*. *pneumoniae* mortality in pups, so effects on clinical parameters between the treated and control cohorts were also investigated. A broader sample of pups were monitored for clinical signs to investigate the course of disease in affected pups. Clinical signs, haematology and oral and rectal swabs to detect gastrointestinal carriage of hypervirulent *K*. *pneumoniae* were not useful for detection of disease prior to death. Of those pups that died due to hypervirulent *K*. *pneumoniae*, only 26.1% (18/69) had any clinical signs prior, likely a reflection of the peracute course of disease. On comparison of haematological parameters between ivermectin-treated and control pups, significantly lower total plasma protein and higher eosinophil counts were seen in control versus treated pups, however standard length as a surrogate for age was a more important influence on parameters overall than ivermectin treatment. This study also highlighted a cohort of pups with severe clinical signs suggestive of hypervirulent *K*. *pneumoniae* infection were lost to follow up at the end of the monitored season, which could be contributing to cryptic juvenile mortality.

## Introduction

Septicaemia due to a hypervirulent (HV) variant of an otherwise common enteric bacterial commensal, *Klebsiella pneumoniae*, is the primary cause of death in New Zealand (NZ) sea lion (*Phocarctos hookeri*) pups at Enderby Island, Auckland Islands in the NZ sub-Antarctic [1]. The pathogen became endemic at this site after emergence during an epizootic event in 2001–02 and whilst multiple threats likely contribute, the impact of HV *K*. *pneumoniae* on pup mortality has been ranked amongst the most significant factors affecting survival at a population scale for the species [2]. Demographic modelling predicted that alleviation of HV *K*. *pneumoniae*-associated pup mortality would result in positive population growth for the species, a predicted outcome that modelled abatement of other threats including commercial trawl-related mortality and trophic effects causing food limitation did not achieve [2]. Initial work has demonstrated deworming pups with the anthelmintic ivermectin at around one week of age reduces the risk of HV *K*. *pneumoniae* mortality, likely by preventing intestinal mucosal damage by hookworms and therefore likelihood of systemic dissemination [3]. Following prolonged population decline and then stabilisation at lower pup production estimates at the Auckland Islands onwards from 2013–14, a Threat Management Plan was developed to facilitate targeted research and investigate potential mitigation strategies aimed toward abating and reversing the population decline of the species [4]. To that end, risk factors for pup mortality have been examined to determine targets for preventative mitigation in order to reduce disease in individual pups [3], but an alternative approach could involve developing efficient early detection, diagnosis and treatment of pups with HV *K*. *pneumoniae* disease.

Whilst the gross and microscopic pathology of HV *K*. *pneumoniae* mortality in NZ sea lion pups have been reported in relative detail [1, 5, 6], the clinical course of disease is poorly described in the literature. Reports emanating from the initial epizootic appearance of disease in 2001–02 at Enderby Island described a marked increase in the number of dead pups found in the first two months post-partum, and moribund pups identified with fluctuant limb swellings, lameness, lethargy and terminal convulsions [7]. Other than this, no published reports describe morbidity of pups with HV *K*. *pneumoniae* disease and therefore it is not known whether pups deteriorate so acutely that it is not obvious to observers before a moribund state, or if pups are able to recover following clinical signs of infection. Further, if any treatment were to be undertaken, diagnosis would need to be possible in a remote field situation. Therefore, we aim to characterise the clinical course of *K*. *pneumoniae* disease in NZ sea lion pups, including the range of clinical signs, haematological response to infection and detection of the pathogen in the gastrointestinal tract. As this work was undertaken concurrently with an ivermectin clinical treatment trial, and given the protective effect of ivermectin treatment on HV *K*. *pneumoniae* mortality [3], an additional aim of the study was to define the haematological response in ivermectin-treated and control pups.

## Materials and methods

All work was undertaken in the greater Sandy Bay area (50.5°S, 166.28°E), Enderby Island, Auckland Islands during the 2016–17 and 2017–18 austral summer field seasons. Three studies were undertaken concurrently in a nested design throughout both seasons: a case-control study where cases were dead pups and controls were randomly-selected live pups [3], a randomised controlled clinical trial with ivermectin, and a prospective cohort study (‘cohort study’) to investigate risk factors for pup mortality (S1 Fig). Whilst data collected from pups during all studies are included, the latter two components were most relevant to understanding clinical parameters and ivermectin treatment for the current investigation.

Procedures were permitted by the Department of Conservation, New Zealand (necropsies 39239-MAR, all other procedures were permitted as species management). All methods were approved by the Massey University Animal Ethics Committee (approval number 16/89) and Department of Conservation Animal Ethics Committee (approval number 304).

### Animal selection and sampling

Selection for the cohort study was randomised at the first capture when pups were approximately one week of age by enrolling every sixth and third pup in 2016–17 and 2017–18 respectively until n = 50 and n = 100 were attained, as determined by power analysis. Selection for the ivermectin treatment trial was also randomly assigned using a random number table for every pup captured, with independent randomised selection to the cohort groupings [3]. Treated pups were administered 0.2mg/kg ivermectin subcutaneously into the interscapular region on a single occasion at first capture. Pups were individually identifiable after this first capture by a passive integrated transponder (PIT) tag, a temporary vinyl numbered disc attached to their rump or after mid-January by permanent flipper tags (Jumbo Tag, Dalton Continental, Lichtenvoorde, Netherlands) [3]. Cohort study pups were then recaptured approximately fortnightly after their first capture for serial sampling. Data from pups not in the cohort study were collected at their first capture, when these animals were randomly captured as controls in the case-control study, when they displayed abnormal clinical findings, or when found dead. All dead pups underwent necropsy as soon as practicable with samples collected and analysed to determine cause of death as described in [1].

At all pup captures, a comprehensive dataset of risk factor information was recorded including spatial, environmental and pup variables [3]. Pups were captured by hand or net and restrained in a custom canvas bag for examination. Following morphometric measurements including body mass and standard length, a full physical examination was undertaken noting ocular, nasal or umbilical discharges, signs of infection at flipper tagging sites, physical abnormalities such as joint swellings, dyspnoea or skin wounds and mentation. On release of pups at the site of capture, gait and balance were assessed subjectively. Oral and rectal swabs were collected to determine the presence of HV *K*. *pneumoniae* and faeces collected to identify presence or absence of hookworm ova [3]. Additional to this, cohort study pups also had rectal temperature measured at each capture using a digital thermometer, and during the 2016–17 season only, up to 5mL whole blood was also collected from the brachial vein. Pups were manually restrained in lateral recumbency with the pectoral flipper extended to visualise the medial surface. The skin was disinfected with 0.5% chlorhexidine (Microshield 5, Schulke, Auckland, New Zealand) and venepuncture undertaken with a 21G x 1” needle and 5mL syringe (BD, Auckland, New Zealand). Blood was stored in 1.3mL EDTA and plain serum tubes (Sarstedt, Nümbrecht, Germany) and kept cool in an insulated bag until processing.

### General colony monitoring

The Sandy Bay colony was monitored by researchers during daylight hours between early-December and early-March for resights of individual animals, detection of dead pups and those with abnormal physical or behavioural findings. From the beginning of the field season until pup tagging in mid-January, daily colony counts of pups and females were attained by calculating the mean of at least three direct counts of the colony.

Throughout the 2017–18 field season only, weekly pup censuses were additionally undertaken after pup tagging to determine which individual pups were present and those that had likely emigrated from the Sandy Bay region. This involved a three-person team moving through the extent of sea lion habitat in the greater Sandy Bay area and recording the identity (flipper tag and/or PIT tag) of all pups encountered. The weekly census provided additional opportunities to identify pups with abnormal physical or behavioural findings amongst the population.

### Field laboratory processing

Blood samples and oral and rectal swabs were initially processed in a field laboratory, within six hours of collection. EDTA anticoagulated blood was used to make blood smears that were air dried and fixed in 100% methanol prior to transport back to Massey University, New Zealand. Additional blood smears were made after incubating a 1:2 solution of new methylene blue stain and EDTA blood for ten minutes at room temperature, for later reticulocyte estimation. Whole EDTA-preserved blood was collected into haematocrit tubes and centrifuged at 6000 rpm for five minutes to determine packed cell volume (PCV; %). Total plasma protein (TPP; g/L) was measured by handheld refractometer. Oral and rectal swabs were transferred to cryovials (Cryo.S™ 2mL, Greiner Bio-One, Frickenhausen, Germany) and frozen in liquid nitrogen prior to return to Massey University, New Zealand where they were stored at -80°C until analysis.

### Laboratory processing

Blood smears were stained with Leishman’s stain (Amber Scientific, Midvale, WA, Australia) for the determination of estimated white blood cell counts and differentials performed at a commercial veterinary pathology laboratory (New Zealand Veterinary Pathology, Palmerston North, New Zealand). Erythrocyte abnormalities were recorded on a standard scale based on number of cells affected per high power field (not present, mild, moderate, marked). Reticulocyte counts were performed on new methylene blue stained blood smears using the Miller ocular reticle technique [8], concurrently at the same laboratory.

Oral and rectal swabs were cultured for detection of *Klebsiella* spp. initially using differential media (CHROMagar™ Orientation, Fort Richard Laboratories, Auckland, New Zealand) as described in [3]. Resultant pure cultures were prepared into an ethanolic suspension for microbial identification by matrix assisted laser desorption/ionisation-time of flight mass spectrometry (MALDI-TOF). Representative consistent colonies were subjected to the string test, with isolates capable of producing a viscous string >5mm considered to be hypermucoviscous (HMV) [9] and in this case were used phenotypically to reflect the clonal HV *K*. *pneumoniae* lineage on Enderby Island [10].

### Evaluation of clinical signs

Clinical signs and abnormalities detected in individual pups that could be consistent with HV *K*. *pneumoniae* infection were categorised into four major groups: neurological signs, lameness and/or joint swelling, ocular abnormalities and non-specific signs. Where individual pups had clinical signs in more than one category, the summary category was assigned according to the most severe or extensive signs (S1 File). Specific neurological signs included tremors, twitching, myoclonic seizures, obtundation, ataxia, opisthotonos or head tilt. Lameness and/or joint swelling encompassed reports of pups moving stiffly, limping or dragging one or more limb and/or diffuse or focal swelling of a limb. Abnormalities associated with the eyes included blepharospasm or cloudy opacity in either the cornea or anterior chamber. Ophthalmic examination equipment was not available in the field so further characterisation was not possible in live pups. Healthy NZ sea lion pups are generally very mobile, gregarious, readily interact with other pups, and quickly respond to their mother’s call with movement in that direction or return calling (pers. obs). Therefore, non-specific signs were recorded when pups did not appear ‘healthy’ on observations and encompassed reports of lethargy, shivering, poor responsiveness, separation from other pups or reluctance to move to a mother’s call, when other specific clinical signs were not noted.

### Statistical analysis

Clinical sign duration before death between categories was compared with the Mann-Whitney test. The chi squared test (*χ*^2^) was used to determine associations between categorical variables and 95% confidence intervals for proportions were calculated using the prop.test function in R (Version 3.6.0) [11]. Linear mixed effects models were run using the lmer function in the lme4 package [12] for the outcome of each haematological parameter with covariates of sex, standard length and ivermectin treatment all with individual pup identity as a random effect. Principal component analysis was performed for haematological parameters and primary factors including standard length and ivermectin treatment using the prcomp function and plotted using the ggbiplot package in R [13]. For all analyses, p <0.05 was considered the threshold for statistical significance.

## Results

**Fig 1:**
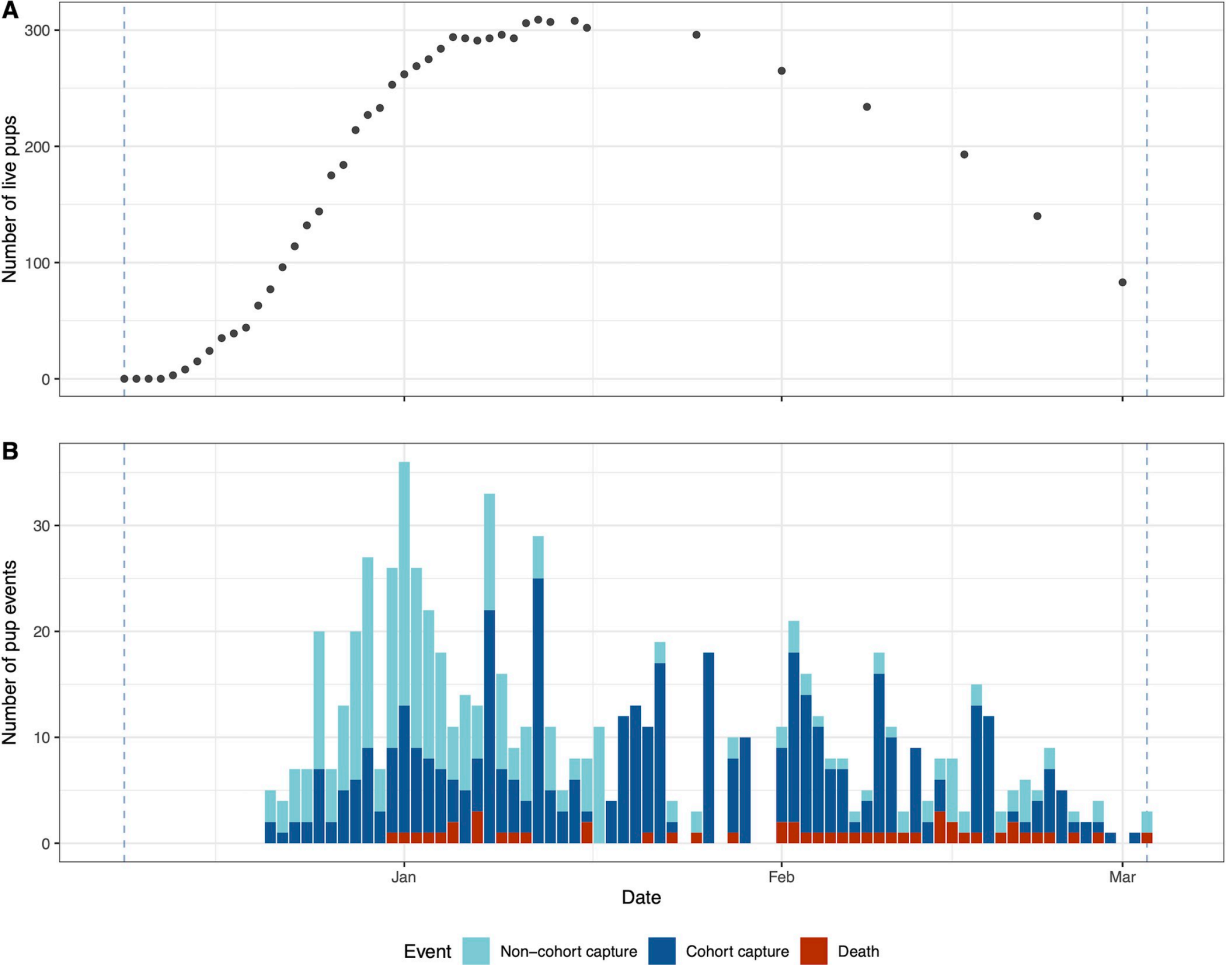
Summary of New Zealand sea lion pup census and events at Sandy Bay, Enderby Island during the 2017–18 season. Vertical dashed lines indicate the beginning and end of the monitored period. (A) Estimated cumulative pup count during daily colony monitoring until mid-January and then weekly census counts ongoing. Decreasing numbers of pups from mid-January are predominantly due to dispersal away from monitored areas. (B) Number of pup captures and deaths per day. Non-cohort captures include first and case-control captures.

Overall, 698 pups were included in the three studies over the 2016–17 and 2017–18 seasons [3], but not all took part in the ivermectin treatment trial due to dying prior to recruitment or alternative colony of origin. These additional animals could be considered as supplemental controls but are not included in the following analyses due to limited individual data collection. Across both seasons, the overall number of pups at Enderby Island recruited to the treatment trial and administered with ivermectin (n_T_) was 343 and the number of pups recruited as controls (n_C_) was 323 (S1 Fig). The prospective cohort study consisted of 150 pups (n = 50, 2016–17; n = 100, 2017–18) at recruitment, with related serial captures totalling 153 and 391 in 2016–17 and 2017–18 respectively, not including further captures (n = 31) where pups were randomly selected as controls in the case control study [3]. The cohort study mirrored the near even selection of treatment groups (n_T_ = 74, n_C_ = 76). Recruited cohort animals were captured a median of three times in 2016–17 and four times in 2017–18, with the weekly pup census from mid-January onwards in 2017–18 (Fig 1) reflecting progressive dispersal of pups away from the site, which reduced the number of pups available for recapture by the beginning of March to 27% (83/308) of the mid-January peak. This emigration also limited follow up of clinical or moribund suspected HV *K*. *pneumoniae* cases as the season progressed. Over both monitored seasons, a total of 124 pups died (17.8%). Of these, 108 were pups recruited to the ivermectin treatment trial (n_T_ = 37, n_C_ 71; 2016–17 n = 62, 2017–18 n = 46); however only 27 (21.8%; n_T_ = 6, n_C_ = 21; 2016–17 n = 14, 2017–18 n = 13) had clinical signs detected prior to being found dead. When only HV *K*. *pneumoniae* cases were considered, of the 69 pups that died, (63 of which had been recruited to the ivermectin treatment trial (n_T_ = 17, n_C_ = 46; 2016–17 n = 36, 2017–18 n = 27)), only 18 (26.1%; n_T_ = 3, n_C_ 15; 2016–17 n = 10, 2017–18 n = 8) had clinical signs observed prior to death.

### Clinical signs

Throughout both seasons and including all pups at Sandy Bay, 50 pups were detected with clinical signs: neurological signs (n = 7, S1 Video), lameness and/or joint swelling (n = 27, S1 Video), ocular abnormalities (n = 5) and non-specific signs (n = 9). Two emaciated pups had non-specific clinical signs including lethargy and poor responsiveness that were determined to be due to their moribund state and were excluded from further analysis.

**Fig 2:**
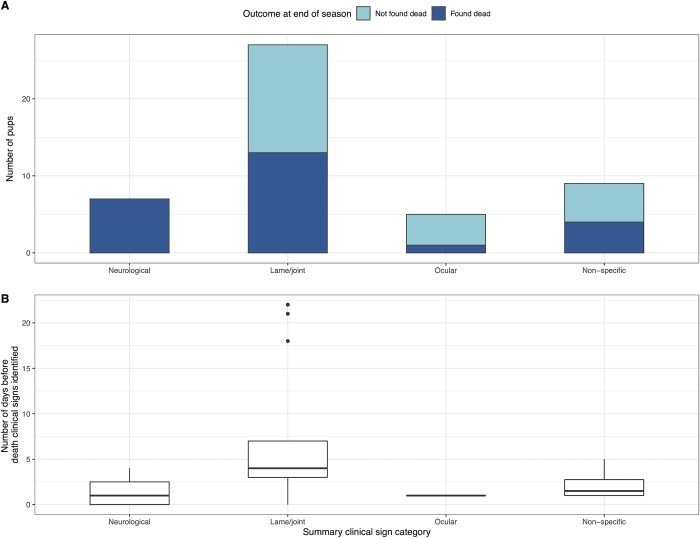
Summary of clinical signs observed in New Zealand sea lion pups at Enderby Island during the 2016–17 and 2017–18 field seasons. (A) Outcome of pups observed with clinical signs during the monitored field season. (B) Number of days before death that clinical signs were first identified, if the pup was found dead.

Of pups with observed clinical signs, 25 (52.1%) were subsequently found dead (Fig 2A). Pups with neurological (n = 7, median 1 day, interquartile range (IQR) 0–2.5 days, range 0–4 days; p = 0.02) or non-specific (n = 4, median 1.5 days, IQR 1–2.75 days, range 1–5 days; p = 0.049) signs died significantly faster than those with lameness and/or joint swellings (n = 13, median 4 days, IQR 3–7 days, range 3–22 days; Fig 2B). The single pup with ocular abnormalities that died, was found one day after observation of clinical signs.

#### Neurological signs

All pups with observed neurological signs died, and all except one (85.7%, 6/7) were diagnosed with HV *K*. *pneumoniae* as the cause of death. The remaining case was a pup with a grossly evident congenital skull malformation and associated subdural haemorrhage determined on necropsy.

#### Lameness and/or joint swelling

Of 27 pups observed with lameness and/or joint swelling, 13 (48.1%) died during the monitored period, with 12 of these diagnosed with bacterial septic arthritis or cellulitis, of which eight were due to HV *K*. *pneumoniae*. The remaining cellulitis or septic arthritis cases (n = 4) were caused by *Salmonella* Kottbus, *Streptococcus phocae* and *Psychrobacter sanguinis* (one case confirmed, one case presumed [3]). The remaining case was diagnosed to have died due to trauma and milk aspiration with puncture wounds and swelling of the right tarsal joint noted grossly. Histopathology demonstrated necrosis and active suppurative inflammation at the site but without significant microbial growth on culture.

#### Ocular abnormalities

Five pups had a summary clinical diagnosis of ocular abnormalities, of which one died due to drowning and HV *K*. *pneumoniae* infection, with hypopyon amongst the clinical findings. Of the remaining four cases, one was sighted only once more, three days later and three were not sighted for the remainder of the season after the record of ocular abnormality. One additional pup classified in the neurological category due to clinical sign severity also presented with blepharospasm and died due to HV *K*. *pneumoniae* septicaemia with hypopyon confirmed at necropsy.

#### Non-specific signs

Nine pups were recorded with non-specific signs, of which four were found dead. All four died due to bacterial infections (n = 3 HV *K*. *pneumoniae*, n = 1 histological signs of infection but no isolate cultured [1]). The HV *K*. *pneumoniae* cases displayed a combination of consistent necropsy findings including septic polyarthritis, pyothorax, suppurative meningitis and subdural haemorrhage, one also with hypopyon. The case with no isolate cultured had severe cellulitis of the right antebrachium with septic arthritis of the right shoulder. The pup had a grossly swollen right carpus at necropsy, but the pup was only reported clinically prior to death as lethargic and isolating from other pups.

An additional 23 pups with clinical signs were lost to follow up (presumed dispersal to or death in an unmonitored area). Fourteen of these pups had lameness or joint swelling, five had non-specific signs and four had ocular abnormalities. Many had severe clinical signs consistent with those preceding death in other animals, followed by either the pup being observed to leave Enderby Island by sea with its mother or simply by no further sightings.

### Haematology

#### Utility of haematology to detect hypervirulent *Klebsiella pneumoniae* infection

Due to the fortnightly interval in blood sampling of cohort pups during the 2016–17 season, only 16.7% (5/30) of pups that died were sampled within three days of their death (n = 2 within 2 days, n = 3 within 3 days). Only two of these died due to HV *K*. *pneumoniae* (sampled two and three days before death), and neither had total or differential white cell counts outside reported ranges for clinically healthy otariid neonates [14–16].

Band neutrophils were only noted in nine blood smears from eight pups, five of these pups did not have band neutrophils on subsequent captures, one was lost to follow up early in the season but was not found dead, one died three days after sampling [3]. The final pup died twelve days after sampling due to drowning and HV *K*. *pneumoniae* infection. This pup was also the only pup in which toxic changes to segmented neutrophils were occasionally noted in the blood smear.

#### Haematology and ivermectin treatment trial

**Fig 3:**
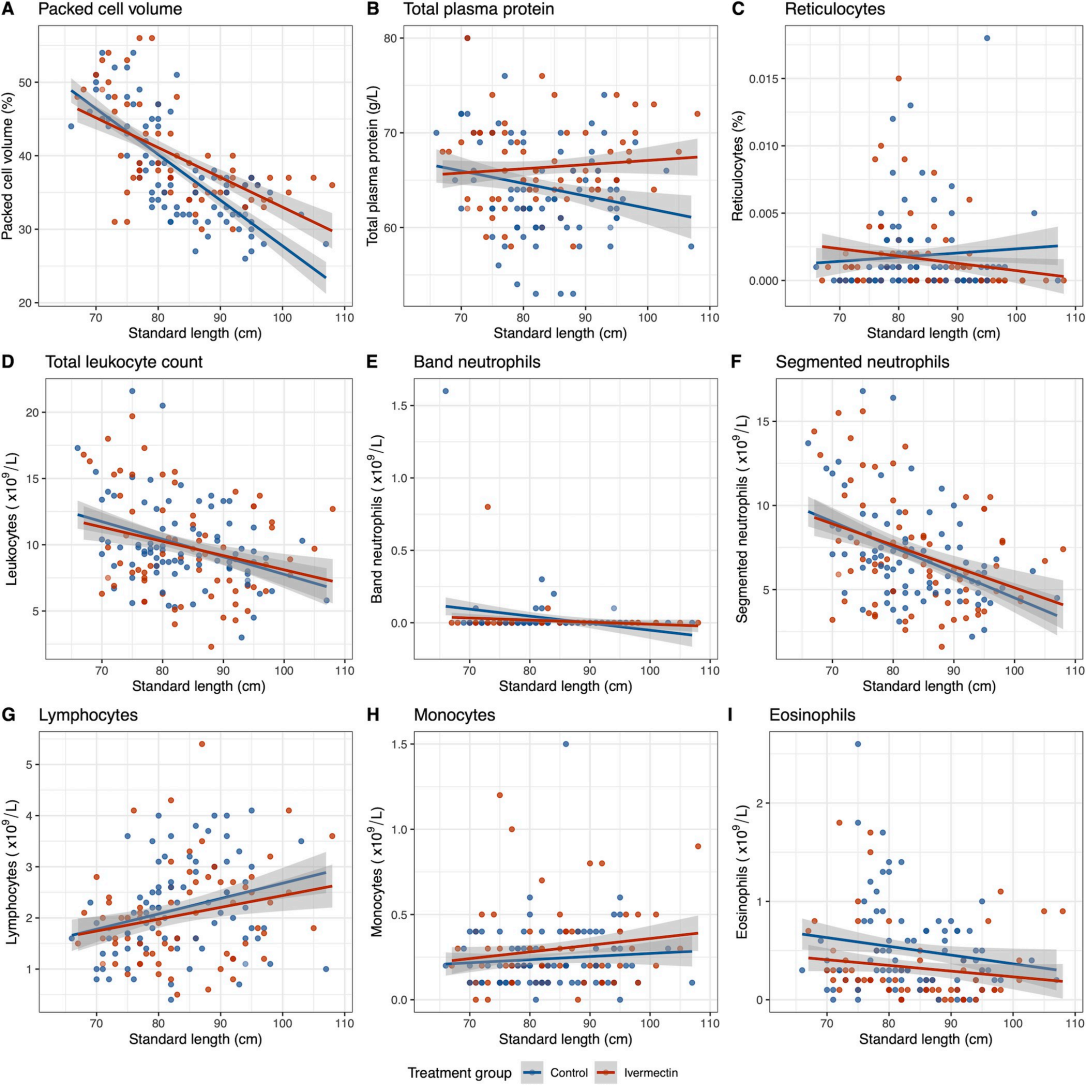
Line plots showing change in haematological parameters with standard length as a surrogate for age and ivermectin treatment group in New Zealand sea lion pups. Grey shading indicates 95% confidence intervals. Haematological parameters include (A) packed cell volume, (B) total plasma protein, (C) reticulocytes, (D) total leukocyte count, (E) band neutrophils, (F) segmented neutrophils, (G) lymphocytes, (H) monocytes, (I) eosinophils.

The results of linear mixed models analysing haematological parameters of serially sampled cohort study pups with covariates of standard length, sex and ivermectin treatment group are summarised in Table 1 and Fig 3. Standard length as a surrogate index for age [3] significantly affected several variables, with packed cell volume, total leukocyte count, absolute segmented neutrophil and eosinophil counts decreasing, while absolute lymphocyte count increased with age. Ivermectin-treated pups had significantly higher TPP concentrations and significantly lower absolute eosinophil counts compared to control pups (Table 1), but treatment group did not significantly influence any other haematological parameters. Sex did not significantly influence any haematological variables. Reticulocytes were not suitable for analysis due to low variance within the sample. A principal component analysis incorporating haematological parameters, standard length and ivermectin treatment group, showed the strongest principal component only explained 31.9% of the variance, with no clustering evident (S2 Fig).

**Table 1:** Summary of linear mixed effects models for haematological parameters of New Zealand sea lion pups with covariates of standard length as a surrogate for age, sex and ivermectin treatment group, with individual pup identity as a random effect. Sample size for ivermectin-treated (n_T_) and control (n_C_) pup samples are listed.

	n_T_	n_C_	Intercept Estimate (SE)	Standard length Estimate (SE)	Sex (Male) Estimate (SE)	Ivermectin Estimate (SE)
Packed cell volume (%)	83	70	85.52 (3.69)	-0.58 (0.04)***	1.57 (1.07)	1.69 (1.05)
Total plasma protein (g/L)	83	70	67.39 (3.62)	-0.031 (0.044)	-0.91 (0.90)	2.01 (0.87)*
Total leukocyte count (x 10^9^/L)	82	70	21.04 (2.43)	-0.14 (0.029)***	0.62 (0.71)	-0.08 (0.69)
Band neutrophil count (x 10^9^/L)	82	70	0.015 (0.008)	1 x 10^−4^ (9 x 10^−5^)	-6 x 10^−4^ (0.003)	-0.002 (0.003)
Segmented neutrophil count (x 10^9^/L)	82	70	19.73 (2.06)	-0.16 (0.02)***	0.47 (0.62)	0.15 (0.61)
Lymphocyte count (x 10^9^/L)	82	70	0.036 (0.69)	0.024 (0.01)**	0.15 (0.16)	-0.10 (0.16)
Monocyte count (x 10^9^/L)	82	70	0.02 (0.16)	0.003 (0.002)	-0.034 (0.042)	0.047 (0.041)
Eosinophil count (x 10^9^/L)	82	70	1.17 (0.33)	-0.01 (0.004)*	0.06 (0.07)	-0.17 (0.07)*

***, P<0.001

**, P<0.01

*, P<0.05; SE, standard error

Erythrocyte abnormalities occasionally noted on blood smear evaluation included anisocytosis, polychromasia, poikilocytosis, echinocytes, eccentrocytes and codocytes. With multiple univariate analyses, anisocytosis, polychromasia and poikilocytosis were significantly associated with lower PCV however ivermectin treatment was not a significant influence. Basophils were not observed in any of the blood smears examined.

### Hypermucoviscous *Klebsiella pneumoniae* carriage

Oral swabs were significantly more likely to culture positive (7.1%, 97/1371, 5.8–8.6% 95% CI) for HMV *K*. *pneumoniae* than rectal swabs (5.1%, 69/1357, 4.0–6.4% 95% CI; *χ*^2^ = 4.39, p = 0.036). This is despite relatively even detection of all *K*. *pneumoniae* (HMV and non-HMV) on oral (22.6%, 310/1371, 20.4–24.9% 95% CI) and rectal swabs (23.9%, 325/1357; 21.7–26.3%; *χ*^2^ = 0.61, p = 0.43) overall. On 40 captures of 36 individual pups, both oral and rectal swabs were positive for HMV *K*. *pneumoniae*.

Of the 150 pups monitored throughout the prospective cohort study, 52 (34.7%; n_T_ = 25, n_C_ = 27) were positive for HMV *K*. *pneumoniae* as determined by oral or rectal swabs at least once. Thirty pups from the cohort died (20%, 30/150; n_T_ = 7, n_C_ = 23), of which 19 (63.3%, 19/30; n_T_ = 1, n_C_ = 18) died due to HV *K*. *pneumoniae*. Of these dead pups, three (15.8%) had a positive oral or rectal swab prior to death and 15 (79%) had a positive swab at necropsy. In comparison, 34 (22.7%, 34/150; n_T_ = 22, n_C_ = 12) cohort pups that were not found dead, had an oral or rectal swab test positive from at least one capture.

### Rectal temperature

There was no significant difference between rectal temperature of cohort pups at an individual capture and ivermectin treatment group or total leukocyte count in univariate analyses. The highest temperature recorded (>40°C, beyond the limits of detection of the thermometer), was measured in a pup with a markedly swollen hindlimb (S1 Video; pup ID G089) that died two days later and was diagnosed with septic polyarthritis, hindlimb abscessation and cellulitis due to HV *K*. *pneumoniae*. Due to the proximity of the primary lesion to the rectum, it is difficult to determine if the elevated temperature was reflecting local inflammation, systemic pyrexia or both.

## Discussion

This study, focussing on morbidity rather than mortality in HV *K*. *pneumoniae* cases in NZ sea lion pups, is a novel focus in the literature. Diagnostic indicators of infection such as clinical signs, physical examination findings, haematology and positive cultures of oral and rectal swabs were not practical tools for the detection of HV *K*. *pneumoniae* disease in NZ sea lion pups at Enderby Island. Given the known variation in lesions and body systems affected following HV *K*. *pneumoniae* bacteraemia [1], variability in clinical signs and blood parameters would be expected, however a large proportion of pups died without any detectable premonitory signs. Whilst this could indicate insufficient intervals in pup sampling and monitoring for disease detection, which was certainly the case for haematology analysis, clinical signs were surveyed at least daily across the colony, so death due to HV *K*. *pneumoniae* without indicative clinical signs prior, likely reflects the peracute course of disease. Rapid deterioration with high mortality rate is well recognised in neonatal septicaemia in domestic animal species [17, 18] and even with a vast swathe of tests available in a clinical setting, a combination of diagnostic tests and patient factors has been shown to have more prognostic predictive value than a single test alone [19, 20]. Without the diagnostic applications available in a controlled environment, such as blood culture with bacterial identification, definitive pre-mortem diagnosis of bacteraemia or septicaemia is difficult. Further, on a remote island working with free-ranging wildlife and in challenging conditions, even repeat monitoring of individual patient clinical signs is problematic. With this in mind, this study confirms that the limited diagnostic capacity present on Enderby Island is unlikely to be useful at a large scale to detect sub-clinically ill pups that may be amenable to treatment or supportive care.

Whilst the presence of neurological signs in NZ sea lion pups justified a high index of suspicion for HV *K*. *pneumoniae* central nervous system disease, they were only observed a median of one day prior to death. Conversely, most pups that died due to HV *K*. *pneumoniae* had central nervous system lesions at necropsy [1], but were not observed to display neurological signs before death. Lameness and/or joint swelling were detectable in some cases for a prolonged period but did not result in confirmed death as frequently as neurological signs. Only 48% (n = 13) of pups detected with lameness or joint swelling were found dead and the cause in those cases, while often bacterial in aetiology, was only due to HV *K*. *pneumoniae* in 62% (n = 8) of necropsy cases. Outcomes in this category were complicated by several factors. Firstly, lameness without joint or limb swelling was not detectable unless the pup was mobile, reducing the ability of observers to detect cases. Secondly, variability in prognosis associated with lameness would be influenced by the pathogenesis of the lesion which is not externally evident. For example, a pup with a single joint infection caused by extension into the joint from an adjacent subcutaneous lesion may have a better prognosis and longer clinical course than a pup with septic polyarthritis secondary to bacteraemia. Pups with lameness and/or joint swelling represent the largest cohort of potentially HV *K*. *pneumoniae*-affected pups that could die following the conclusion of the monitored season, but their actual outcome is not known. Non-specific clinical signs were by definition, variable in duration and outcome, but this category was included to indicate a ‘sick’ pup without other obvious findings. Rectal temperature was also of limited use to detect HV *K*. *pneumoniae* infection in NZ sea lion pups. This could be a consequence of few captures of cohort pups in the perimortem period and study design did not allow for capture and full examination of pups that showed suspicious clinical signs, although pyrexia is also not a consistent finding in calf septicaemia [18, 19].

Whether pups can recover once clinical signs are present was challenging to determine; however, all pups detected with neurological signs in this sample subsequently died. Resolution of bacterial meningitis, septic arthritis and septicaemia is unlikely even with veterinary intervention. Human cases of systemic HV *K*. *pneumoniae* infection, even with access to hospital treatment, are associated with significant morbidity and mortality [21]. Treatment is required for weeks to months and those patients that survive may develop long term complications such as blindness [22] or amputation due to osteomyelitis [23]. These treatments are not feasible or compatible with survival in free-ranging NZ sea lion pups. It should also be considered that any prolonged period of pup debilitation may result in maternal abandonment, such that even if pups could eventually recover, they would likely then go on to starve.

An ongoing challenge in understanding the dynamics, risk factors and clinical effects of HV *K*. *pneumoniae* in this mobile gregarious marine mammal is the species’ natural dispersal throughout the breeding season and beyond. Whilst the early stages of dispersal are somewhat predictable, with movement from Sandy Bay beach after pupping onto surrounding sward and southern rātā (*Metrosideros umbellata*) forest, later dispersal to other sites around Enderby Island and beyond make serial captures and follow up of individual animals a near impossible task. Consequently, survival of juvenile NZ sea lions (between 2–3 months and 3 years) is a major knowledge gap, other than being modelled as low [24, 25]. This is mediated by poor resightability between the end of the field season in which pups are born, until the progressive return of breeding age animals (>4 years) at the natal site for breeding and pupping. As a result, whilst HV *K*. *pneumoniae* is the primary cause of death in the pup age class at Enderby Island, the effects in older pups and juveniles are unknown as researchers cannot easily access this age cohort for monitoring. The high number of pups in this study that had severe clinical signs at their last sighting indicates that a potentially significant proportion of HV *K*. *pneumoniae* mortality likely occurs following dispersal and could contribute as cryptic mortality to the poor juvenile survival rate. The dearth of information on the juvenile age class remains a key source of uncertainty regarding mortality, survival and dispersal in the species which may impact demographic modelling outcomes [24].

Haematology was not useful as a screening tool to detect HV *K*. *pneumoniae* disease. In this study, the approximately fortnightly interval in serial sampling during the 2016–17 season was designed to sample a large cohort of pups, but resulted in fewer samples per pup, such that the likely period of clinical disease was not captured for most pups that died. The two pups that were sampled several days prior to death due to HV *K*. *pneumoniae* did not display haematological abnormalities. Even in well monitored domestic species, haematology parameters are variable, with both neutrophilia and neutropenia consistent with different stages of systemic bacterial disease [18]. In the current study, total white blood cell count decreased significantly in association with age, a finding also reported in Steller sea lion (*Eumetopias jubatus*) pups [16]. Further work would be required to clarify any relationship between decreasing total white cell count with the onset of HV *K*. *pneumoniae* infection in NZ sea lion pups, however many factors likely influence disease susceptibility, including potential failure of passive transfer of HV *K*. *pneumoniae*-specific antibodies [26].

Several studies have compared haematological parameters of otariid pups between ivermectin-treatment and control groups to assess the clinical effects of hookworm infection [27–29]. Not all studies analysed all parameters, but findings have not agreed; reporting no significant difference between treatment groups for all parameters tested [27], higher erythrocyte counts and lower eosinophil counts in treated pups [28], or higher haemoglobin concentrations in treated pups only when compared with pups with severe hookworm infection [29]. Whilst these studies did not all consider finer temporal changes, baseline haematology modelling in the current study showed that age was a more important influence on most parameters than ivermectin treatment. Only TPP and eosinophil count were significantly different on linear modelling between ivermectin treatment groups, likely mediated by local damage and inflammatory response to the presence of parasites in the intestine.

Packed cell volume significantly decreased with age in both treatment groups for the monitored period, likely associated with maturation of foetal erythrocytes into adult form [30]. Whilst a similar ‘physiologic anaemia of infancy’ is seen in some terrestrial animals including humans [30, 31], the effect in pinnipeds can be more pronounced as the animals adapt from foetal exposure to decreased oxygen levels during maternal foraging, then wholly terrestrial in the neonatal period, to spending increasing time periods foraging in the marine environment [32]. This transition has been reported in neonatal pinnipeds of several species [14, 16, 33], but in comparison to phocids that wean quickly with rapid conversion to adult values, otariids may have a prolonged low PCV as erythrocyte parameters only increase with the development of diving ability for self-sustaining foraging: from five months of age in Steller sea lions [34] and up to one year of age in Galapagos sea lions [32]. The precise advent of adult PCV values has not been identified in NZ sea lions due to only the first three months of life being monitored in pup haematological studies (current study; [27]), but a similar trend likely exists. Whilst it was suggested that the decrease in erythroid parameters in neonatal Australian sea lions were primarily attributable to patent hookworm infection rather than a physiological process [15], in further work in the same species, PCV did not significantly differ in samples of ivermectin-treated and control pups, although total erythrocyte counts did [28]. In the current study PCV decreased at a similar rate in both ivermectin-treated pups and controls, albeit slightly and non-significantly slower in treated pups. Growth rate was also not significantly different in NZ sea lion pups treated with ivermectin [3]. These findings, together with lack of association between reticulocyte counts and ivermectin treatment, reflect either the low clinical relevance of regenerative anaemia and hookworm infection, or lower pathogenicity of the parasite in the species at a broad scale. Additional parameters such as haemoglobin concentration, nucleated erythrocyte and total erythrocyte counts should be assessed to investigate this further. Individual pups may become clinically anaemic, however only one of five pups with PCV <30% (all untreated); had pale oral mucus membranes. This pup had the lowest PCV recorded (26%) but survived to at least the next season (pers. obs).

Detection of oral or rectal colonisation by HMV *K*. *pneumoniae* is also unlikely to be a useful diagnostic test, not least since this can only be detected retrospectively at the end of the season after samples have been transported back to the laboratory. Further, of those cohort pups that died, it was more common to have a positive oral or rectal swab at necropsy than at a routine capture prior to death. So, even if on-island testing were feasible, more frequent swabbing would be required to detect carriage prior to death, which would be impractical compared to the relative conservation management value of testing, however more research is required to understand the temporal dynamics of HV *K*. *pneumoniae* carriage. Whilst a correlation between HV *K*. *pneumoniae* carriage and death due to HV *K*. *pneumoniae* septicaemia was confirmed in NZ sea lion pups at Enderby Island [3], further work is required to demonstrate whether the isolates from oral and rectal swabs, match those from infective lesions as has been reported in human cases [35, 36]. Interventions in humans, including testing and isolation of *K*. *pneumoniae* colonised patients and increased hygiene was associated with a statistically significant reduction in colonisation and bacteraemia [37]. While similar measures would be impossible in wildlife, it does highlight the contribution of environmental contamination to the dynamics of disease.

In conclusion, this work suggests that the peracute course of HV *K*. *pneumoniae* infection in NZ sea lion pups results in a lack of clinical and haematological evidence of illness before death, severely hampering early diagnosis. Pups with severe clinical signs have a grave prognosis and are likely to die before treatment interventions could take effect. Mitigation and positive population-level outcomes are therefore more achievable through manipulation of known risk factors including removal of hookworms by treatment with ivermectin. Finally, the finding that pups with clinical signs of HV *K*. *pneumoniae* infection were lost to follow up at the end of the season requires further exploration to establish whether this could be a significant component of cryptic juvenile mortality in the species.

## Supporting information

S1 Fig Summary of study design and sample size in the three studies to investigate clinical parameters in New Zealand sea lion pups at Enderby Island: Case-control study, ivermectin clinical treatment trial and prospective cohort study. (TIF)

S2 Fig Principal component analysis of major haematological parameters, standard length as a surrogate for age and ivermectin treatment in New Zealand sea lion pups at Enderby Island, 2016–17. (TIF)

S1 Video Summary video of clinical signs in the neurological and lameness and/or joint swelling categories consistent with hypervirulent *Klebsiella pneumoniae* disease in New Zealand sea lion pups at Enderby Island. (MP4)

S1 File List of New Zealand sea lion pups involved in the study with associated information on clinical signs detected. (CSV)

S2 File List of New Zealand sea lion pups involved in the study with associated demographic, haematology and microbiology information. (CSV)
