# Supplementary figures and images for: Clinical parameters of hypervirulent Klebsiella pneumoniae disease and ivermectin treatment in New Zealand sea lion (Phocarctos hookeri) pups
Source: PLoS One. 2022 Mar 3;17(3):e0264582. doi: 10.1371/journal.pone.0264582 (PMC8893627; doi:10.1371/journal.pone.0264582)

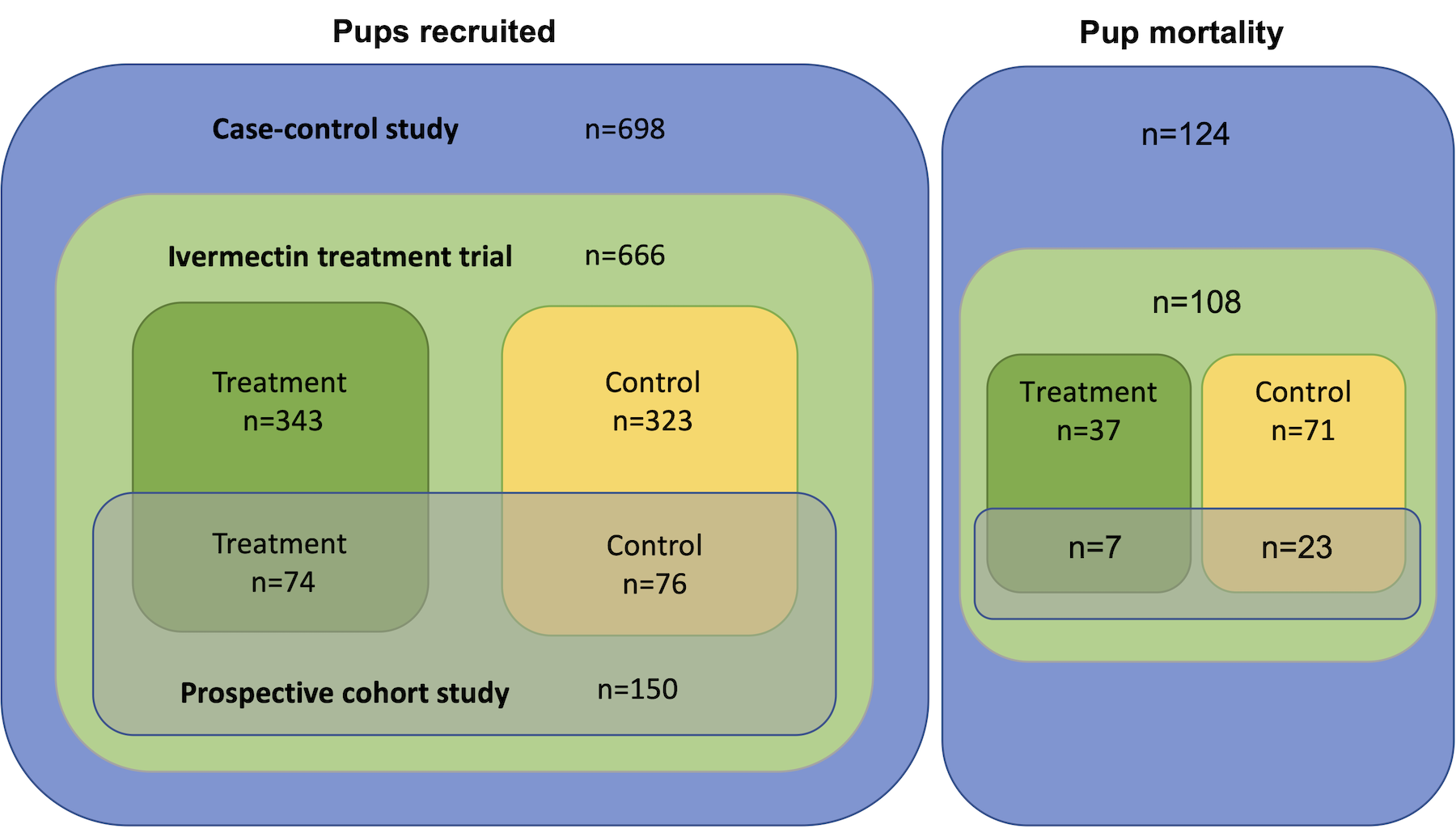

Supplement: S1 Fig — (TIF) [file pone.0264582.s001.tif]

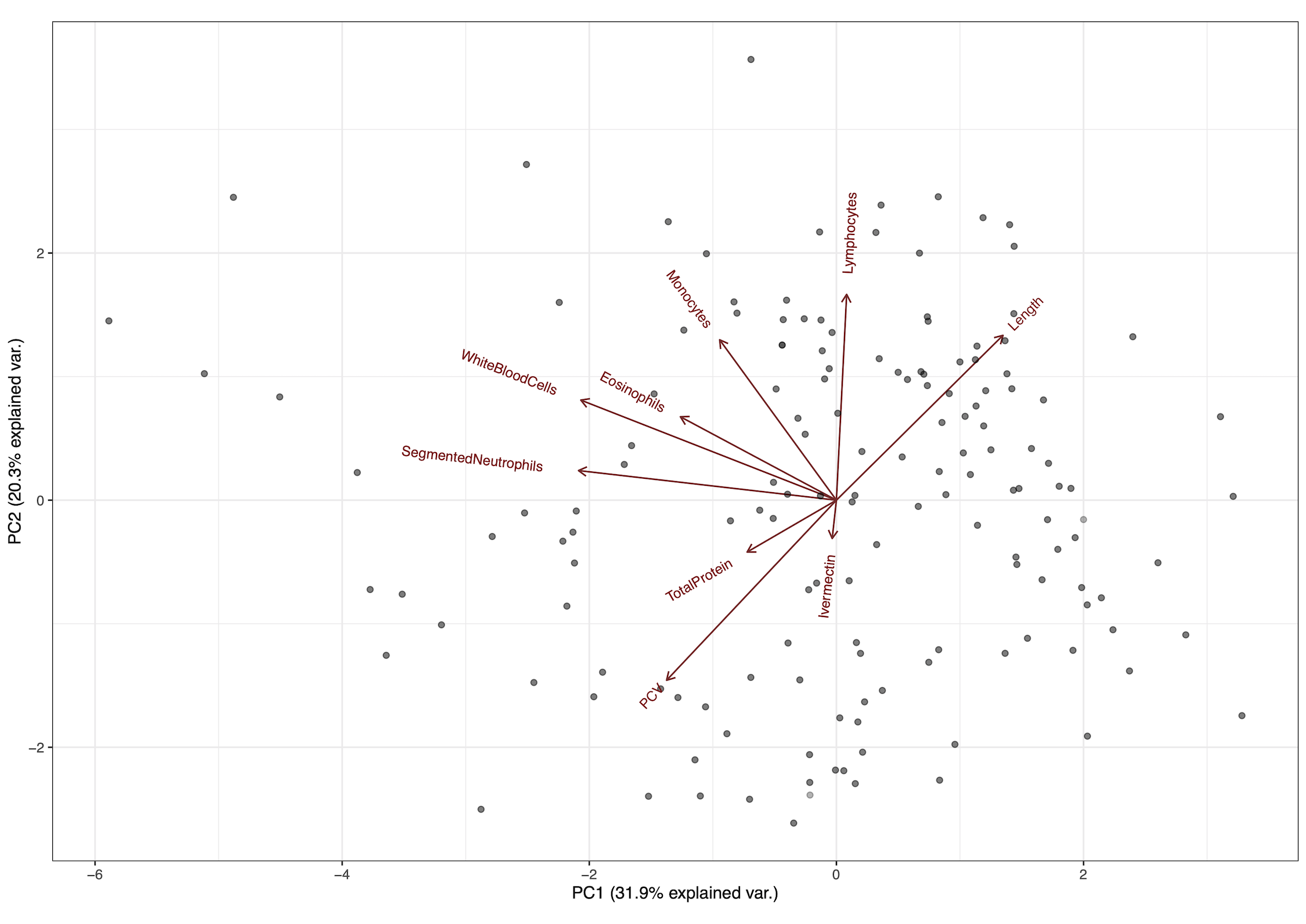

Supplement: S2 Fig — (TIF) [file pone.0264582.s002.tif]
